# The Conceptual Structure of the Management by Nurses of the Ego Integrity of Residents of Nursing Homes

**DOI:** 10.1097/jnr.0000000000000394

**Published:** 2020-06-05

**Authors:** Sun-Young LIM, Sung Ok CHANG

**Affiliations:** 1 PhD, RN, Visiting Professor, College of Nursing, Baekseok Culture University, ROK; 2 PhD, RN, Professor, College of Nursing, Korea University, ROK.

**Keywords:** ego integrity, hybrid model, nursing home

## Abstract

**Background:**

The number of older people admitted to nursing homes has continued to rise with the recent expansion of the Republic of Korea's long-term care system. Maintaining ego integrity is a major task for older people approaching the end of life. As efforts to maintain ego integrity include the final stages of life, this concept is critically important for older people in nursing homes. This study was designed to assess issues related to ego integrity in the nursing home environment to determine how nurses should play a key role in managing this important life task.

**Purpose:**

The management by nurses of the ego integrity of residents of nursing homes is a new phenomenon that is central to promoting long-term, quality care. This study was designed to clarify and conceptualize this management phenomenon in the context of nursing homes.

**Methods:**

A hybrid model of concept development was used to analyze the ways in which nurses manage the ego integrity of residents of nursing homes. In the theoretical phase, a working definition of the management by nurses of residents' ego integrity is developed using a literature review. In the fieldwork phase, in-depth interviews are conducted with eight nurses from six nursing homes in Seoul and three other provinces. Finally, in the final analytical phase, the theoretical and fieldwork findings are interpreted and compared.

**Results:**

Two components, assessment and intervention, of the approach by nurses to managing the ego integrity of residents of nursing homes were identified. Assessment incorporates 10 attributes in the following three dimensions: “identifying the extent to which residents' basic needs are being fulfilled,” “determining how residents achieve friendly relationships with others,” and “determining how each resident creates a harmonious view of his or her life.” Intervention incorporates nine attributes in the following two dimensions: “helping residents develop a positive view of life” and “helping residents make the best use of their remaining functional abilities.”

**Conclusions/Implications for Practice:**

By managing the ego integrity of residents, nurses have a significant influence on residents' sociopsychological adaptation, especially in the challenging environment of a nursing home. This study supports that managing the ego integrity of residents of nursing homes is an important and practical component of the role played by nurses and of the aid and care they provide. Furthermore, the findings verify the effectiveness of intervention studies in examining assessment tools and developing guidelines for ego-integrity management.

## Introduction

Population aging introduces new healthcare challenges such as adding complexity to the medical environment and transferring social care for diversely functional older adults from home and medical facility settings to nursing homes (Kojima, 2015). As more older adults are living longer with geriatric illnesses, nursing homecare is becoming more prevalent (Paek et al., 2016).

The proportion of the population in South Korea aged 65 years or older is expected to increase from 14.3% in 2018 to 41% in 2060 (Korean Statistical Information Service, 2018). To accommodate this aging society, the number of nursing homes has increased significantly since Korea implemented long-term care insurance in July 2008.

As life expectancy continues to increase, efforts have been made to highlight the positive aspects of an aging society, including concepts related to successful aging, gerotranscendence, and ego integrity (Martineau & Plard, 2018; Westerhof et al., 2017;Wong & Yap, 2016). Successful aging suggests sociopsychological completeness in healthy older adults who are able to maintain relatively healthy physical and cognitive functions in the community setting (Chodzko-Zajko et al., 2009), gerotranscendence implies a positive and continually evolving change in the worldview of older people (Wong & Yap, 2016), and ego integrity refers to positive sociopsychological adaptation in older adults.

As nursing homes require that residents engage in sociopsychological adaptation, practitioners have used ego integrity to induce positive adaptation in nursing home settings (Erikson, 1963; Lim & Chang, 2018a; Torges et al., 2008). Nursing home practitioners manage the ego integrity of residents by helping them establish a more harmonious and positive relationship with their caregivers (Torges et al., 2008). The management by nurses of residents' ego integrity relies on nursing-specific role characteristics, which are useful for ensuring quality care (James & Zarrett, 2006; Westerhof et al., 2017).

Ego integrity, or the integration of life cycles for older people that embraces death, is recognized as something that even older people who are unhealthy or near to death may pursue. Eco integrity emphasizes a traditional focus on establishing relationships with others, aiming for a mature and harmonious life until the very end of life. Thus, there is a growing need to promote ego integrity in older people who are spending the last days of their lives in nursing homes. In fact, the management of their ego integrity is a part of nurses' responsibilities and, thus, must be clearly identified to organize nursing care services properly, to promote continuity of interventions, and to check on the effects of interventions. As nurses are in a position to evaluate the physical, sociopsychological, and mental status of residents, they are in an optimal position to design personalized care and play an important role in managing residents' ego integrity. Thus, residents' quality of life in their final months and the quality of nurse/resident relationships may be improved.

Previous studies of ego integrity have focused primarily on older adults living in the community (Dezutter et al., 2016; Jo & Song, 2015; Tahreen & Shahed, 2014). Few studies have explored ways of helping older adults make this transition and integrate into nursing homes. Recently, Lim and Chang (2018a) studied the ways in which nurses perceive ego integrity in residents of nursing homes. They observed that nurses held subjective perceptions and that they should reflect on the concept as part of their care activities. This finding suggests that nurses are well positioned to manage residents' ego integrity effectively. This emerging phenomenon is worth exploring, given that nursing may significantly improve residents' quality of life. This study clarifies and conceptualizes the ways in which nurses manage the ego integrity of residents of nursing homes in their daily practice.

## Methods

### Study Design

This concept-development study adopted the hybrid model, which is used extensively to clarify, create, develop, and extend concepts, particularly in nursing. The hybrid model clarifies concepts to create new and more comprehensive definitions. At times, definitions emerge that differ completely from previous ones. The hybrid model combines inductive and deductive approaches, identifying the essential aspects of a concept and providing clarity through observation and interviews based on actual participant experiences. The main concept is derived (particularly when the data are ambiguous) through integrative literature analysis and fieldwork study (Schwartz-Barcott & Kim, 2000). Thus, the hybrid model consists of three phases: theoretical, fieldwork, and final analysis.

### Data Collection and Procedure

#### Theoretical phase

“Management” refers to the process of dealing with or controlling things or people (Oxford University Press, n.d.). The development of knowledge about nurses' management belongs within the practice domain for enacting assessment and treatment activities (Schwartz-Barcott & Kim, 2000). The concept of nurses' management of residents' ego integrity was developed by dividing this phenomenon into two aspects: assessment and intervention.

**Figure 1:**
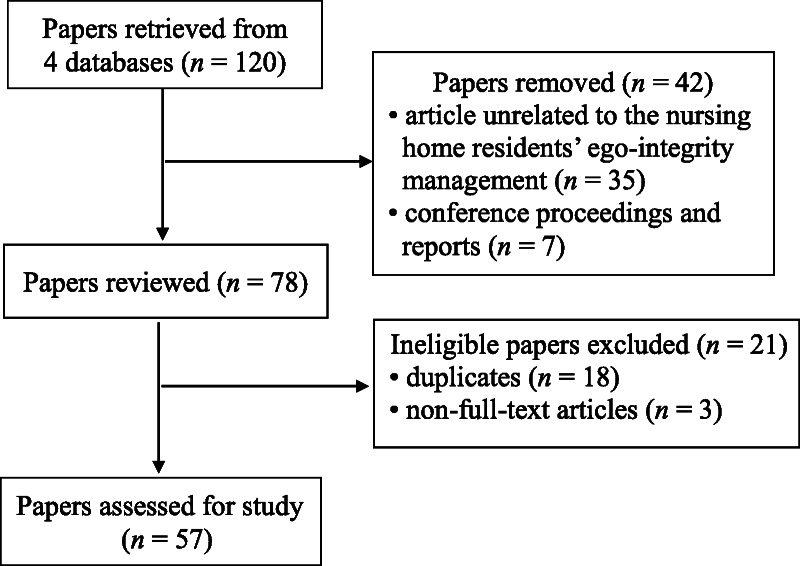
A Flowchart of Data Selection and Data Extraction in the Theoretical Phase

A literature search was carried out using the following keywords: “nursing home,” “nursing-home residents,” “ego integrity,” “management,” “assessment,” “intervention,” and “strategy.” Electronic databases including PubMed, CINAHL, MEDLINE, and ProQuest were searched for articles published between 1963 and March 31, 2018, using the search options provided by each database and EndNote software. Articles were included only if they had at least one keyword in the title, abstract, or list of key words. An initial search yielded 120 articles. After eliminating duplicate titles, 78 articles remained. Of these, 21 articles were excluded, leaving 57 articles available to review (Figure 1), including nine on psychology, three on social welfare, two on education, and 43 on nursing. The dimensions and attributes deemed most significant in articles of a theoretical nature are presented in Table 1. The important issues related to the assessment and intervention for the literature review are as follows. The assessment uncovered meanings of ego-integrity management, which help determine how residents achieve friendly relationships with others and how each resident creates a harmonious view of his or her life. Interventions that were found to relate to managing the ego integrity of residents included those designed to help patients develop a positive view of life and to make the best use of their remaining functional abilities.

**Table 1:** Contents of Major Literature Review From a Theoretical Phase

Study Author (Year)	Conceptual Dimension and the Attribute of Ego-Integrity Management
Lim & Chang (2018a)	Nurses confirmed that, when residents identified new solutions to problems, flexible acceptance of a new lifestyle could be inferred. Here, ego integrity could be assessed through group activities and confirmed through stories retold by other residents. Identifying clues to the residents' positive acceptance of their whole life spans; identifying residents' ways of enjoying their current lives, referencing residents' attitudes toward and ability to form harmonious relationships; and identifying residents' integrated efforts to establish self-esteem. Wisdom helps individuals recognize experiences they can use to reconstruct their lives; this process is akin to the way in which individuals reconcile important social relationships in old age.
Park, Lim, Kim, Lee, & Chang (2018)	To help residents make the most of their remaining functional abilities, nurses must help them adjust to certain internal and external constraints. This could include having residents use spoons whenever possible to maximize their remaining capacities.
Harrison & Frampton (2017)	Identifying the residents' friendly relationships with others allowed nurses to assess how well the residents were adjusting to life in the nursing home.
Friedman et al. (2017)	Strengthening and maintaining high-quality relationships with the residents' families and friends outside the nursing home is another important factor for achieving ego integrity.
Hung et al. (2016)	Reducing problematic behaviors through safe environmental controls is essential. Specifically, it is important to provide a “home-like” atmosphere within the nursing home.
Drageset, Eide, Dysvik, Furnes, & Hauge (2015)	Identifying how residents create a harmonious view of life requires a meaningful self-image. Self-esteem is a key component of this dimension among residents.
Roberts & Bowers (2015)	This leads to good relationships between nurses, families, and other residents. Such relationships have a positive effect on residents' ability to articulate problems and reduce distress.
Haugan (2014)	Helping residents generate a positive view of life was best accomplished by creating calm experiences through everyday comforts. The residents often achieved ego integrity by enjoying small tasks.
Lim et al. (2014)	Harmonious relationships with other residents; the will for self-actualization; the spiritual needs required when actualizing and using ego integrity; the palliative care needed in nursing homes. To help residents accept death objectively as an extension of life, nurses must facilitate transcendental thinking about end-of-life issues. This task illustrates the nature of nursing science in relation to managing a peaceful death.
Vitale, Shaffer, & Acosta Fenton (2014)	The quality of a resident's relationship with his or her family and transcendent thoughts help to actualize ego integrity, leading to successful aging; this is a significant responsibility for nurses.
Brandburg, Symes, Mastel-Smith, Hersch, & Walsh (2013)	It is crucial to improve the residents' quality of life by analyzing and managing their physical, mental, and social status and interacting with them accordingly. Continuously confirming gratitude for life has been shown to be important. Those who positively express this gratitude have integrated the end of life well.
Aleksandrova-Yankulovska & ten Have (2015)	Preparing for the process of ego-integrity actualization and the positive acceptance of death: reflections and a positive attitude toward life and management are important for a positive death.
Ali & Ayoub (2010)	Importantly, spiritual peace and acceptance of a peaceful death are phases of ego-integrity actualization for the residents. Participation in religious activities can often lead to spiritual peace. Such activities can provide social benefits, aid with functional limitations, and assist with managing death anxiety. In the context of terminal care, a peaceful death can be advocated, while residents focus on physical, cognitive, social, and spiritual functions that mediate the integrity of their remaining abilities. In essence, spirituality can contain wider and deeper meanings above and beyond functional recovery or rehabilitation.

#### Fieldwork phase

The participants were eight nurses working at six nursing homes in Seoul and three other provinces. The selection criterion was being a nursing home nurse with more than 3 years of experience since 2008, when the long-term Korean care insurance was enacted and when nursing homes began to be formally evaluated. The participants had professional experience with residents of nursing homes. Before data collection, the director of each nursing institution was contacted to approve the data collection procedures. The participants, all volunteers, signed a written agreement stating that they were willing to be interviewed and could refuse to participate at any time. Interview questions were formulated, and the researcher personally conducted interviews based on the theoretical assessment and intervention areas. Each interview took 1–2 hours, and each participant completed one to three audiotaped interviews.

The interview data were transcribed, coded, and reviewed. Interviews and field observations were transcribed verbatim, and the content of transcripts was analyzed to identify and classify categories relevant to the key research questions. Each transcript was read several times to allow for full immersion. When all of the data had been coded and the categories were condensed, each category was assessed to determine data saturation.

#### Final analytic phase

The results, which reflected the essence of ego-integrity management among residents of nursing homes, were analyzed and compared with results obtained during the fieldwork phase. The key concepts derived from a review of the literature during the theoretical phase and the elements of concepts identified during fieldwork were reexamined, compared, synthesized, and analyzed to arrive at a final definition of ego-integrity management in the context of residents.

### Ethical Considerations

This study was approved by the institutional review board of the authors' institution (Certificate Number: KU-IRB-16-276-A-1), and permission to conduct the research was obtained from each facility. The purpose of the study was explained to all participants, who were informed that their involvement was voluntary. Participants were assured that the information they provided would remain confidential.

## Results

### Theoretical Phase: Review of the Literature

A working definition of the ego-integrity management attributes and dimensions of residents of nursing homes was elicited theoretically using the experiences of the nurses. The concept of ego integrity includes various attributes, qualities, and meanings. Additional concept dimensions were defined by analyzing and classifying attributes. The nurses' management of residents' ego integrity was classified using attributes from the assessment and intervention dimensions.

#### Definition of the concept

The Oxford English Dictionary (2018) defines the ego as “the part of the mind that mediates between the conscious and the unconscious and is responsible for reality testing and a sense of personal identity,” whereas integrity is defined as “the state of being whole and undivided,” and management is defined as “the process of dealing with or controlling things or people” (Oxford University Press, n.d.). Thus, “ego-integrity management” may be defined as intervention and coaching to lead a subject toward a balanced and harmonious goal rather than as a subclassification of the ego.

No precise definition of ego-integrity management currently exists for residents of nursing homes. However, related assessments and interventions have been used with people experiencing profound disabilities, diseases, and injuries (Korean Ministry of Health and Welfare, 2018). In these cases, ego-integrity management has helped subjects accept their lives without remorse, be content with their present lives, have a harmonious view of the past and future, and have no fear of death (Erickson, 1963). In addition, ego-integrity management may refer to the process by which nurses help residents achieve this actualized goal using systematic assessments and individualized interventions. From the perspective of nurses, no precise definition of ego-integrity management exists for residents of nursing homes. Using assessment and intervention, ego-integrity management guides residents into a state in which they do not fear death but rather accept their lives with satisfaction and without regret, experiencing harmony between past, present, and future. The systematic assessment and personalized interventions provided by nurses help residents achieve as much ego integrity as possible.

#### Concepts related to ego integrity

The concepts of successful aging and gerotranscendence are used in similar ways in nursing. Successful aging involves maximizing positive and minimizing negative outcomes. Efforts to organize and integrate one's whole life in old age may be less comprehensive than ego integrity (Rylands & Rickwood, 2001). Gerotranscendence refers to the transformation of an older adult worldview that is universal and rational rather than materialistic and rational (Erikson, 1998). However, neither concept expresses the essence of ego integrity, which refers to positive sociopsychological adaptation in old age.

### Ego-Integrity Management for Residents of Nursing Homes: Assessment

The assessment in this study uncovered two distinct meanings of ego-integrity management: (a) determining how residents achieve friendly relationships with others (with four attributes) and (b) determining how each resident creates a harmonious view of his or her life (with three attributes).

#### Determining how residents achieve friendly relationships with others

Attributes include residents' flexibility in accepting new ways of life, their active acceptance of others' ways of life, the strengthening of social relationships using the transition process, and wisdom based on accumulated experience.

The first step in identifying residents' social relationships is assessing how well residents have adjusted to life in the nursing home (Harrison & Frampton, 2017). Nurses have confirmed that, when residents come up with new solutions to problems, flexible acceptance of a new lifestyle may be inferred. Here, ego integrity may be assessed through group activities and confirmed through stories retold by other residents (Lim & Chang, 2018a).

A resident's social relationships may be strengthened within the new context even before admission to a nursing home. Afterward, emotional bonds emerge through communication and contact with others living in the facility (Björk et al., 2017). The maintenance of quality relationships with family and friends outside the nursing home (Friedman et al., 2017) and lifetime wisdom, based on accumulated experience, both help actualize ego integrity among residents. Wisdom helps individuals recognize experiences that they may use to reconstruct their lives. This process is akin to the way in which individuals reconcile important social relationships in old age (Lim & Chang, 2018a).

#### Determine how each resident creates a harmonious view of his or her life

Attributes for this dimension include residents' abilities to recognize themselves as individuals, their acceptance of death as an extension of a worthy life, and their continuous confirmation of gratitude. To create a harmonious view of life, residents must envision their own self-image as meaningful. Self-esteem is a key component of this dimension (Drageset et al., 2015). Residents must also accept the inevitability of death and frame it as an extension of a worthwhile life. To those living life as an extension of the past, accepting the reality of their current situation may bring comfort and fulfillment (Meier et al., 2010). Furthermore, continuously confirming gratitude for life has been shown to be important. Those who positively express this gratitude have integrated end of life well (Brandburg et al., 2013; Lim & Chang, 2018a).

### Ego-Integrity Management for Residents of Nursing Homes: Intervention

Interventions reveal two reasons for managing the ego integrity of residents: (a) to help them develop a positive view of life (with four attributes) and (b) to make the best use of their remaining functional abilities (with four attributes).

#### Helping residents develop a positive view of life

Attributes included inducing a feeling of calmness through everyday comforts, which led to good relationships with nurses, family members, and other residents. Residents also found it easier to accept their past lives and view death as an extension of life.

The best way to help residents generate a positive view of life is to create calm experiences within a comfortable and satisfying living environment. For instance, residents often achieve ego integrity by carrying out small tasks (Haugan, 2014; Lim & Chang, 2018a). This feeling may lead to quality relationships that have a positive effect on residents' abilities to articulate problems and reduce their own distress (Roberts & Bowers, 2015). To help residents accept death objectively as an extension of life, nurses must facilitate transcendental thinking about end-of-life issues. This task illustrates the nature of nursing science in relation to managing a peaceful death (Ali & Ayoub, 2010; Lim et al., 2014). Finally, a comprehensive acceptance of the past helps residents reconcile their successes and failures, which fosters wisdom (James & Zarrett, 2006).

#### Helping residents make the best use of their remaining functional abilities

To develop this dimension, nurses encourage residents to use their remaining physical capacities, promoting increased mobility, spiritual comfort, and stability. Moreover, nurses guide residents to perceive their environment as safe.

To help residents make the most of their remaining functional abilities, nurses help residents adjust to internal and external constraints. For example, residents use spoons whenever possible to maximize their remaining capacities (Park et al., 2018). Movement may also be encouraged even among residents who are bedridden. Nursing homes in the United States have adopted “function-focused care” and “functional restorative care” in accordance with regulations for maintaining the functional capacities of residents who are bedridden. These interventions aim not only to maintain functional abilities but also to make these abilities last as long as possible. They serve as a method of assessing physical functioning within a nursing home context (Boltz et al., 2011; Resnick et al., 2007).

Participation in religious activities often leads to spiritual peace. Such activities may provide social benefits, ameliorate functional limitations, and assist with managing death anxiety. In the context of terminal care, a peaceful death may be advocated, while residents focus on physical, cognitive, social, and spiritual functions that mediate the integrity of their remaining abilities. In essence, spirituality contains wider and deeper meanings that are above and beyond functional recovery or rehabilitation (Ali & Ayoub, 2010; Lim et al., 2014). Reducing problematic behaviors through safe environmental control is a key factor. Specifically, it is important to provide a “home-like” atmosphere in nursing homes (Hung et al., 2016) to help residents feel as safe and autonomous as possible. Home-like interiors and music have been shown to reduce residents' problematic behaviors (Amella et al., 2008).

Management by nurses of the ego integrity of residents of nursing homes has been defined in the literature as follows.

**A working definition of the process of managing the ego integrity of residents:** In the theoretical phase of this study, nurses were found to manage the ego integrity of residents in ways that could be categorized into assessment and intervention components. A working definition of this process must include ways of determining whether a resident is showing friendly relationships with others through flexible acceptance of a new way of life and active acceptance of others' new ways of life, strengthening social relationships, and showing wisdom through accumulated experience. This definition must include the ways in which residents create a harmonious view of life, recognize that they are worthy people, accept death as an extension of a worthy life, and continuously confirm gratitude. The definition should also recognize that residents seek peace through life comforts; maintain good relationships with nurses, family members, and other residents; accept death positively; and accept their own life. Finally, ego integrity should be developed through interventions that help residents make the best possible use of their remaining functional abilities, encourage the use of physical capacities and increased mobility, promote spiritual comfort and stability, and guide residents to maintain a safe environment.

### Fieldwork Phase

During the fieldwork phase of the assessment, the literature was reviewed to elicit a working definition of managing the ego integrity of residents of nursing homes. In addition, a new dimension was added. During the intervention phase, the first dimension (helping residents develop a positive view of life) was derived from one theoretical attribute. During the fieldwork phase, each attribute was broken down into a detailed guide to the meaning of residents' interpersonal relationships, particularly with regard to family members.

The assessment and intervention components are outlined, respectively in Tables 2 and 3, indicating how attributes were revealed in detail by nurses during the fieldwork phase. This section describes a novel dimension that was newly discovered during the assessment and presents aspects in a new way.

**Table 2:** Summary of Major Assessment Dimensions and Attributes From the Fieldwork Phase

Dimension/Attribute	Content Described	Relevant Quotation From Interviews (Nurse No.)
**1. Determine how residents develop friendly relationships with others**		
(a) Residents' flexible acceptance of a new way of life	According to the nurses, residents who showed no resistance to living in an unfamiliar environment and got along well with others achieved a comfortable daily routine by positively adapting to the environment (i.e., through more smiles and positive facial expressions).	“*When an older adult has an easygoing attitude, it's clear that he or she is adapting better. Making friends in a nursing home is so critical, especially when you are not feeling so well, for the friends you make are going to be your family. Those who have a good relationship with others are often more content with their lives.*” (Nurse 6)
(b) Residents' active acceptance of others' ways of life	Residents who think of others first, as well as listening to and helping others, tend to have better relationships with others, showing thoughtful consideration.	“*There are residents sharing the same room with individuals with severe Alzheimer's disease, who cannot eat without having food falling out of their mouths. However, those residents help clean such individuals, even before we ask and call for residents who have been lying in bed for too long. They even celebrate each other's birthdays and give gifts.*” (Nurse 2)
(c) Residents' strengthening social relationships	Residents who are usually self-regulated are constantly striving not to disturb others. They try not to complain, listening to (and respecting) others for who they are. This helps residents build stable relationships with those around them.	“*There are some residents who are concerned about many friends and family members. These residents show gratitude by saying how much they appreciate the help they get when their diapers are changed. That really warms our hearts.*” (Nurse 3)
(d) Residents' wisdom, based on accumulated experience	Residents' wisdom is usually revealed through their attitudes or actions. Those with wisdom often are more considerate and build quality relationships. A solid interpersonal organizational style that reflects wisdom gained over one's life is an important factor when assessing ego integrity.	“*When their families are not able to visit often, some residents do not seem to be upset. Instead, they just have more small talks with their roommates or take the lead in having a fun time. Surprisingly, they tend to call their families to tell them they are really having a good time, are having no trouble with eating or sleeping, and the family does not have to visit the nursing home if they are too busy.*” (Nurse 4)
2. Determine how residents create a harmonious view of their own lives		
(a) Residents recognizing themselves as worthy people	When the residents look back on their lives, they value their volunteer jobs, make efforts to build a better life, and respect their accomplishments and what they wanted to do, instead of having regrets and hatred. Those are the criteria for a meaningful life mentality, with which they judge their own ego integrity.	“*Some residents have helped others who are weak and not as materially sufficient, and that is what made their life meaningful. That kind of thought is really where grace comes from. For instance, there is a charity organization called ‘The Green Umbrella.’ We have a resident who's been a member of that organization since 1948, and now her son and grandson are actively volunteering in the organization.*” (Nurse 4)
(b) Residents' acceptance of death as an extension of a worthy life	The acceptance of death is shown by residents overcoming anxiety and developmental crises surrounding death; this helps to determine the residents' quality of life.	“*‘I will not have any regrets, even if I die while I am sleeping tonight.’ Those who say things like that are really the ones who have lived their lives to the fullest; you can really feel the weight of their words. Also, even when Cheyne–Stokes respiration occurs during a resident's final hours, the peaceful facial expression explains everything.*” (Nurse 3)
(c) Residents' continuous confirmation of life gratitude	The residents who show gratitude whenever a visitor or staff member helps are the embodiment of gratitude. Even when a resident was born in a difficult time, he or she is still grateful for living a positive life.	“*There are some residents who really show their gratitude everywhere. They thank the physical therapist for the comfort provided and thank other staff members for their work, appreciate the nursing home program, and even stay till the end to help clean up an area after an activity. They really do not hold back on their gratitude.*” (Nurse 8)

**Table 3:** Summary of Major Intervention Dimensions and Attributes From the Fieldwork Phase

Dimension/Attribute	Content Described	Relevant Quotation From Interviews
1. Help residents adopt a positive view of life		
(a) Help residents experience calm through the comforts of life	The nurses intervene with the residents by helping them recognize seasonal changes and what the residents do best and enjoy. Eventually, they emphasize composure and acceptance of death.	“*Helping residents realize this ‘season of change’ is really effective. We help them feel this change by showing different pictures or changes to the garden art. Showing them movies can really enrich their cultural life. When they see some classic movies starring Audrey Hepburn, whose grace is widely appreciated, their memories about the good old days come across.*” (Nurse 2)
(b) Help residents accept death objectively as an extension of life	The nurses realize that accepting death in the continuum of life is critical when intervening to foster ego integrity. Especially when dying well is considered, combining the idea of death with religion or emotions to make the acceptance of death easier for each individual is an important factor when intervening to foster ego integrity.	“*We often see that residents of the same religion find peace when singing or listening to spiritual songs together. They also seem to accept death more peacefully, even when they are just talking about their religion.*” (Nurse 6)
(c) Help residents accept their past lives	According to the nurses, when residents have regrets or negative attitudes about the past, it is important to help them accept the past through a positive intervention, which allows residents to live the rest of their lives (and accept death) more peacefully.	“*As most of the residents had difficult lives, the way they think about their past affects how they accept death. There are some residents who changed their attitude toward the past through reminiscence therapy.*” (Nurse 3)
2. Help residents make the best use of their remaining functional abilities		
(a) Encourage the use of remaining physical abilities	The nurses emphasized the importance of allowing residents to use their remaining physical functional abilities with the least amount of help possible.	“*It's better for us to wait for the residents to use utensils and eat by themselves. That allows the residents to feel better about themselves, for they can still eat using their hands at will. Things they can do with their hands, like using the remote controller to change the TV channel and using a hand fan for 10 minutes as a punishment for losing a roshambo game, are helpful. It's also good to give the residents wet wipes to let them clean up themselves.*” (Nurse 1)
(b) Encourage increased mobility	The nurses emphasized to residents that movement, considering a resident's level of mobility in bed, is important for helping residents use their remaining functional abilities.	“*We try to let the residents use their remaining functional abilities; however, for those who cannot leave their beds, we encourage them to use the bedside rails to help them get up, lie down, or rotate. In order to maintain their remaining physical functional abilities, we also train residents to use their hips when changing positions. For ladies with severe hemiplegia, we suggest that they try their best to manipulate their own legs.*” (Nurse 2)
(c) Help residents perceive a safe environment	The nurses emphasized that residents should perceive their environment as safe and comfortable, similar to a home, so as to adapt to the new environment faster.	“*It's important to make the residents feel at home. That's why we ask their families to bring things the residents really appreciate from home. There are some residents who enjoy folk songs, so we ask their families to bring folk-song cassette tapes for the residents to listen to.*” (Nurse 4)
(d) Promote spiritual comfort and stability	The nurses said that it was important to assess each resident's spiritual needs and help him or her find spiritual comfort and stability.	“*There's no emotional bond greater than religion. However, we need to assess each resident’s spiritual needs instead of just putting them all together, as if they had the exact same religion to begin with. Helping residents find their own spiritual comfort and stability, individual by individual, is the key.*” (Nurse 8)

### Ego-Integrity Management for Residents: Assessment

#### Identify the extent to which residents' basic needs are fulfilled

Minimal self-care meets a resident's basic physiological needs. According to the nurses, residents who could manage their basic needs for defecation, urination, toothbrushing, eating, and dressing and who wanted to engage in self-care were more likely to achieve positive self-realization and self-esteem outcomes.

There are times when the residents try to perform self-care. For example, even with difficulties, they still insist on eating or brushing their teeth by themselves. This reminds me of Maslow's Hierarchy of Needs Theory. If basic physiological needs are not met, one cannot achieve higher level needs such as those in the emotional, intellectual, and spiritual dimensions. These older adults often show a higher level of self-esteem and are more tolerant of others, which is part of those higher-level needs.

**Psychological acceptance of self-management limits:** Residents who accept the reality of their limitations without frustration and ask for help when they need it show better psychological acceptance of ego integrity, leading to a more comfortable attitude toward death.

I think those who accept their limits have a better chance of adapting their ego integrity, as they demonstrate better emotional control, showing no anger or impatience even when the situation is aggravating.

**Willingness to engage in physical activity:** According to the nurses, residents who show willingness to participate actively in everyday physical activities (e.g., cooperating during diaper changes and during transfers from bed to wheelchair) are more contented and accept the present reality in a more positive way.

Some residents cooperate when we try to transfer them from the bed to a wheelchair for a walk instead of just staying still. Even though they cannot move exactly as they want anymore, the will to actively participate in everyday physical activities makes them feel content.

### Ego-Integrity Management for Residents: Intervention

#### Helping residents develop a positive view of life

**Helping residents discover meaning in interpersonal relationships:** According to the nurses, relationships with staff members, other residents, and visiting friends play an important role in supporting the residents' ego integrity. Helping residents maintain these relationships is a key feature of the nurses' ego-management activities.

We suggest that the residents smile at their roommates once in a while even when having to stay in bed. Getting along with people around them is critical for alleviating loneliness and depression, which is what they need to do to build good relationships with others. You cannot feel the same amount of happiness as when you smile together with others when you just smile alone.

**Helping residents construct meaningful relationships with family:** The nurses felt that helping residents in this way was an important intervention to support their ego integrity.

We always try our best to help the residents maintain good relationships with their families, while not forgetting the fact that we (nurses) are also a part of their family at the nursing home. We always suggest that residents take the medicine their families have bought, complete the physical therapy their families want them to complete, and tell their families how everything is going during visits.

The fieldwork phase produced a working definition of ego-integrity management. This definition included helping residents meet their basic needs (including minimal self-care to meet physiological needs, psychological acceptance of their own self-management limits, and willingness to engage in physical activity), maintain positive relationships with others, create a harmonious view of life, and make the best use of their remaining functional abilities.

### Final Analytic Phase

**Figure 2:**
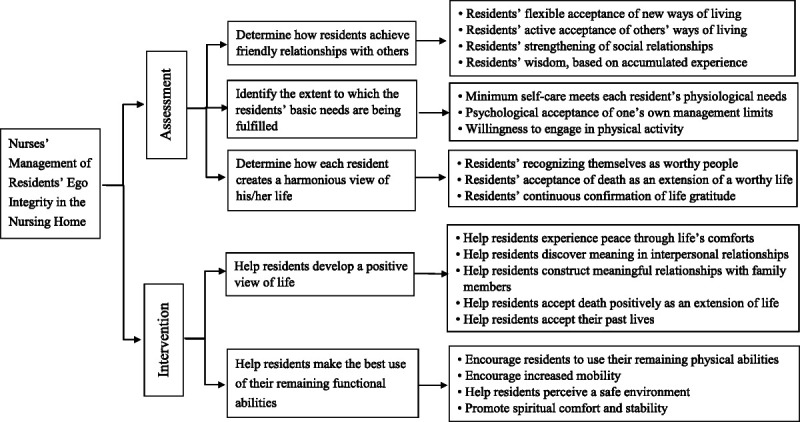
Concept of Nurses' Management of Residents' Remaining Functional Abilities

During the final analytic phase, the nurses' management of ego integrity was assessed across the theoretical and fieldwork phase results, distinguishing between attributes (Figure 2). First, the management concept was developed during the theoretical and fieldwork phases. Next, the dimensions and attributes of each phase were derived. The theoretical-phase results were defined more clearly during the fieldwork phase. The final analysis was performed after attributes from the theoretical phase were sufficiently saturated in the fieldwork phase.

In the final analytic phase, the theoretical and fieldwork results were compared to redefine and establish the attributes of ego-integrity management by nursing home nurses.

#### Definition of ego-integrity management, carried out by nursing home nurses

The ways in which nurses managed the ego integrity of residents were defined as follows: The nurses' management of the residents' ego integrity included the following tasks: determining the extent to which residents' basic needs (such as minimum self-care) were fulfilled; understanding both the residents' psychological acceptance of their own self-management limits and their willingness to engage in physical activity; and determining whether the residents had friendly relationships with others, could flexibly accept new ways of life, could actively accept others' way of life, could strengthen social relationships through a new life process, and actualize wisdom through accumulated experience. This definition also describes how residents worked to create a harmonious view of their whole life's course by recognizing themselves as worthy people, accepting death as an extension of a worthy life, and continuously confirming gratitude.

In terms of interventions, nurses should help residents develop a positive view of life (e.g., by promoting feelings of peace through everyday comforts), discover meaning in interpersonal relationships, construct meaningful relationships with their families, accept death positively as an extension of life, and accept their past lives. Finally, the definition includes helping residents make the best use of their remaining functional abilities by using those abilities to work toward increased mobility. The nurses promote spiritual comfort and stability and help residents perceive their environment as safe.

## Discussion

Sustaining ego integrity is a major task for older adults approaching the end of life (Erikson, 1963) and is a very important concept in nursing homes (Lim & Chang, 2018a). As essential care providers, nurses focus on managing the ego integrity of residents of nursing homes (Brandburg et al., 2013; Roberts & Bowers, 2015). Most previous studies have explored the ego-integrity achievement characteristics of community-dwelling older adults. By contrast, this study assesses ego-integrity issues in a nursing home to determine how nurses play a key role in managing this important life task.

As shown in the findings, managing the ego integrity of residents is different from other concepts related to successful aging that emphasize the positive aspects of old age. Successful aging involves managing sociopsychological integrity, particularly among older people who are healthy and maintain relatively competent physical and cognitive functions (Chodzko-Zajko et al., 2009; Martineau & Plard, 2018). Nurses working in nursing homes offer a different approach to maintaining ego integrity that focuses on relationships with others and helping individuals pursue a mature and harmonious life (Torges et al., 2008; Westerhof et al., 2017). In this context, nurses manage the ego integrity of residents by helping them establish more harmonious and positive relationships with others.

The relevant features of nurses' ego-integrity management were identified but not fully confirmed in the theoretical phase. The fieldwork phase provided insights from actual nursing home nurses and helped to identify key nursing practices (e.g., assessing how residents fulfill basic needs) that sustain the ego integrity of residents. The attributes discovered in this study provide a tutorial on the development of ego-integrity-management training resources.

The theoretical and fieldwork phases confirmed that acceptance of death was part of a continuum that reflected a valuable life—a factor now considered important in palliative care. Acceptance of death does not necessarily mean that a person is prepared for the exact moment of death. Rather, this concept represents the ability to look back on one's past with a positive, peaceful attitude, achieving contentment for the rest of one's life through actualized ego integrity (Kim et al., 2014). This kind of acceptance focuses on physical, cognitive, social, and spiritual functions—all characteristics that underpin peace in palliative care and also highlight the deeper meaning of nursing science (Ali & Ayoub, 2010; Lim et al., 2014).

Previous studies on the ways in which nurses manage the ego integrity of residents of nursing homes have derived concepts exclusively from literature reviews (Lim & Chang, 2018b), a technique that is quite limiting. By contrast, combining a literature review with analytic techniques provides a more comprehensive outline of the dimensions and attributes of a particular concept. The approach of this study was developed to improve both theoretical and fieldwork applicability by constructing a conceptual framework via a combined systematic review and interviews with actual nursing professionals. In this study, features that are highly relevant to the nursing workplace were extracted. By producing a body of nursing knowledge that fits well with current nursing home contexts in South Korea, this study contributes to building a foundation of evidence for an outline of ego-integrity management concepts in nursing home practice.

### Conclusions

Identifying the concept of ego-integrity management in nursing home environments is useful as a way to help residents build quality social relationships and experience fulfilling end-of-life experiences. Ego-integrity management is a useful concept for nursing home practice because it has the potential to improve quality of care for a burgeoning older adult population. According to the findings of this study, strategies for managing the ego integrity of residents are a practical component of the professional, caring role of nurses. This management study focused on the role of nurses in nursing homes. The findings also confirmed the effectiveness of previous intervention studies that examined assessment tools and developed guidelines for ego-integrity management. This study paves the way for developing and implementing interventions for residents of nursing homes by clearly distinguishing the positive aspects of concepts related to ego-integrity management (i.e., successful aging and gerotranscendence).
